# Understanding emotion dysregulation in PTSD – GAD comorbidity

**DOI:** 10.1016/j.janxdis.2025.102985

**Published:** 2025-02-05

**Authors:** Lucy J. Allbaugh, Lucas Marinack, Alison M. Pickover, Abigail Powers, Erica D. Marshall Lee, Marylène Cloitre, Nadine J. Kaslow

**Affiliations:** a Department of Psychology, University of Dayton, Dayton, OH, United States; b Department of Psychology, University of Wyoming, Laramie, WY, United States; c Wave Life Inc., Menlo Park, CA, United States; d Department of Psychiatry and Behavioral Sciences, Emory University School of Medicine, Atlanta, GA, United States; e National Center for PTSD Dissemination and Training Division, Palo Alto VA Health Care System, Palo Alto, CA; NYU Silver School of Social Work, New York, NY, United States

**Keywords:** Emotion dysregulation, Posttraumatic stress disorder, Generalized anxiety disorder

## Abstract

Posttraumatic stress disorder (PTSD) frequently co-occurs with myriad mood and anxiety disorders including generalized anxiety disorder (GAD). Despite this comorbidity’s prevalence, mechanisms underlying the cooccurrence of PTSD and GAD remains understudied. An emotion dysregulation framework routinely is used to understand both PTSD and GAD but has not been applied to the PTSD-GAD comorbidity. Using MANOVA, the present study tested domains of emotion dysregulation (DERS) and of positive emotion regulation (AEQ) as differentiators of PTSD alone versus PTSD with GAD using pre-intervention data from a randomized controlled trial including 292 women with PTSD secondary to interpersonal violence. Five of six emotion dysregulation domains differentiated the two groups: fewer regulation strategies, nonacceptance of emotional responses, impulse control difficulties, lack of emotional awareness, and lack of emotional clarity were associated with comorbidity. Of three positive emotion regulation domains, participants with PTSD alone reported more positive emotionality than those with PTSD and GAD, and those with comorbid PTSD and GAD reported more negative affective interference than those with PTSD only. Rather than specific domains underlying unique presentations, findings indicate a general dysregulation factor, where PTSD-GAD comorbidity is supported by an overall higher level of emotion dysregulation as compared to PTSD alone.

## 1. Understanding emotion dysregulation in PTSD – GAD comorbidity

People with posttraumatic stress disorder (PTSD) often have cooccurring conditions, such as various mood and anxiety disorders; one longitudinal epidemiological study found 91 % of individuals with PTSD carried an additional diagnosis (Bryant et al. 2010). Generalized anxiety disorder (GAD) frequently is comorbid with other diagnoses, including PTSD (Price & van Stole-Cooke, 2015); estimated GAD prevalence among trauma-exposed samples has ranged from 11.1 % to 31.6 % (Bryant et al., 2010; Grant et al., 2008). Despite the prevalence of this diagnostic presentation, GAD’s presence in patients with PTSD is relatively understudied as compared to other conditions comorbid with PTSD (e.g., major depressive disorder) (Grant et al., 2008). Further, the underpinnings of the comorbidity, including mechanisms that underlie both, are poorly understood.

## 2. Nature of the GAD – PTSD comorbidity

GAD comorbidity with PTSD is critical to understand as it is associated with myriad poorer outcomes relative to those with standalone PTSD. First, compared to adults with PTSD alone, those with comorbid PTSD and GAD need higher doses of psychopharmacological medication (Brady & Clary, 2003), experience more chronic symptoms with longer recovery time (Zlotnick et al., 2004), and report greater impairment in functioning (Ginzburg et al., 2010). Comorbidities may complicate treatment by inhibiting the emotional engagement with traumatic material that is required in many effective treatments for PTSD (e.g., Cognitive Processing Therapy; Lloyd et al., 2014); this has been found with respect to social anxiety (Gros et al., 2023), though other findings are mixed (Labbate et al., 2004; Lloyd et al., 2014). GAD may also foster this avoidance, leading to lower treatment uptake and higher rates of dropout (Hembree & Foa, 2000; Miles et al., 2015). No outcomes studies have examined this directly, and perspectives vary with regard to the manner in which PTSD and GAD are thought to be associated. This necessitates a better understanding of the nature of the comorbidity and the shared features of PTSD and GAD, and one way to conceptualize this may be by considering emotion dysregulation domains that underlie comorbid GAD and PTSD.

## 3. Emotion dysregulation in PTSD and in GAD

Some researchers view PTSD and GAD as associated yet distinct (Bryant et al., 2010), while others conceptualize the prevalence of this comorbidity as indicative of an underlying general negative affective cluster (Price & van Stole-Cooke, 2015). Accordingly, emotion dysregulation has been used as a framework for understanding both disorders individually, and may be well suited to understanding their comorbidity. Emotion dysregulation is a transdiagnostic factor linked to the development and maintenance of many types of psychopathology (Kring, 2008), including among those with trauma histories (Weissman et al., 2019), and PTSD specifically (Tull et al., 2007). Persistent negative emotions or aggressive/reckless behaviors in PTSD may reflect underlying problems regulating emotional experiences, while avoidance symptoms of PTSD may reflect unhelpful efforts to regulate emotions that impair adaptive functioning (Powers et al., 2015). Neuroimaging also finds associations between PTSD symptoms and patterns of circuitry complication in areas critical for emotion regulation (Fitzgerald et al., 2018). Emotion dysregulation plays a critical role in the development and maintenance of PTSD symptoms; when parsed from negative affect, the dysregulation - PTSD links remain, thus this association cannot be explained by negative affect alone (Cisler et al., 2010; Tull et al., 2007). Research examining specific dysregulation domains associated with PTSD diagnosis has yielded mixed results, however, and few studies have accounted for comorbid conditions. Reduced emotional clarity, problems with impulse control, limited regulation strategies, and difficulties with goal-directed behavior each have been linked with PTSD diagnosis (Ehring & Quack, 2010; Weiss et al., 2013). Several domains (i.e., lack of acceptance, difficulty engaging in goal-directed behavior, limited impulse control, and low emotional clarity) have been linked with greater PTSD symptom severity (Tull et al., 2007), and lack of emotional clarity has been tied specifically to the hyperarousal and cognitive symptoms of PTSD (Timmer-Murillo et al., 2023).

Emotion dysregulation is a framework also used frequently to understand GAD (Behar et al., 2009); the heightened emotional intensity and reactivity associated with GAD makes it difficult for those individuals to regulate emotions (Mennin et al., 2005). Despite its status as a transdiagnostic factor, overall emotion dysregulation has been positively linked to GAD even when controlling for worry, panic attacks, panic disorder, and severity of depressive and anxious symptoms (Mennin et al., 2005; Tull et al., 2009), suggesting some of these deficits might be uniquely linked to GAD. Fewer studies have investigated specific links between individual domains of dysregulation and GAD, and none have accounted for trauma exposure or symptoms, which is critical given the high rate of comorbidity. However, GAD diagnosis has been linked to specific regulation deficits in emotional clarity, understanding of emotions, acceptance of emotions, ability to engage in goal-directed behavior, and use of regulation strategies (Mennin et al., 2005; Salters-Pedneault et al., 2006). Further, the domain of nonacceptance of emotions has emerged as a meaningful predictor of GAD comorbidity with other anxiety disorders (Mennin et al., 2009), indicating that specific subscales may differentiate between diagnostic presentations. Applying an emotion dysregulation framework to the PTSD - GAD comorbidity may yield important insights, as poor regulatory strategies do appear to increase risk for the development of multiple fear- and anxiety-based disorders (Sloan et al., 2017).

## 4. Emotion dysregulation in the PTSD-GAD comorbidity

Altered regulation is common across myriad disorders (Cludius et al., 2020), but the role of specific domains of dysregulation in maintaining symptoms across diagnoses and within comorbidities is not yet well-established, in part because comorbidities are not routinely accounted for in such studies. In PTSD with comorbid depression, emotion regulation domains were differentially associated with PTSD and depressive symptoms; lack of emotional clarity was associated only with PTSD symptoms (specifically with negative mood and hyperarousal symptoms), while lack of goal directed behavior was solely associated with depressive symptoms (Timmer-Murillo et al., 2023). No studies have tested dysregulation domains as predictors of PTSD alone versus PTSD-GAD comorbidity. Should emotion regulation domains reliably differentiate between these presentations, this information would aid in understanding the genesis of the PTSD-GAD comorbidity and how to best treat it.

Prior work suggests several domains common to both PTSD and GAD, including having limited strategies for regulation, difficulty engaging in goal directed behavior, lower emotional clarity, and lower acceptance of emotions (Tull et al., 2007; Salters-Pedneault et al., 2006; Weiss et al., 2013). One subscale, limited impulse control, has been associated with PTSD but not GAD (Weiss et al., 2013), while lack of awareness of emotions has not been found to be associated with PTSD and specifically has been found not to relate to GAD (Mennin et al., 2005). However, there are few consistencies among studies with respect to what domains cluster together as associated with even one diagnostic category. This might reflect that a general dysregulation domain is a better transdiagnostic predictor than any facet alone (Price & van Stole-Cooke, 2015) or it may be that specific diagnoses are associated with differing clusters of regulatory deficits but when comorbidities are not accounted for and vary across study samples, domains that become attributed to PTSD or GAD become muddied.

## 5. Regulation of positive emotions in PTSD and GAD

Historically, emotion regulation research focused on difficulties regulating negative or unpleasant emotions. However, models increasingly account for the regulation of positive emotions, including positive emotionality, the ability to experience and recognize positive emotions (Carl et al., 2013); hedonic deficit, a lack of positive of pleasurable affect (Watson & Naragon-Gainey, 2010); and negative affective interference, the experience that negative affect interferes with the ability to experience positive or pleasurable affect (DePierro et al., 2017; Frewen et al., 2012). This smaller literature suggests that positive emotion regulation domains may be critical mechanisms in the development and maintenance of PTSD. Positive emotions are experienced as aversive to some individuals with PTSD (Frewen et al., 2012; Wolkenstein et al., 2022). This negative affective interference may heighten impulsivity and reduce behavioral control (Frewen et al., 2012; Weiss et al., 2015). Low acceptance of positive emotions has been linked to PTSD symptom severity (Weiss et al., 2020a), and some PTSD treatment protocols target awareness of positive emotions as a resilience factor linked to recovery (Lane, 2023; Weiss et al., 2020b). Some research also reveals deficits in positive emotion regulation relevant to GAD. GAD may be associated with more awareness of positive emotions; higher awareness of positive emotions has differentiated GAD from other anxiety disorders (Newman et al., 2013).

Given the links to both PTSD and GAD individually, regulation of positive emotions might play an important role in predicting GAD comorbidity with PTSD. Unlike prior work on emotion dysregulation, no domains of positive emotion regulation have been associated with both GAD and PTSD. However, several have been uniquely associated with either GAD (increased awareness of positive emotions) or PTSD (negative affective interference and low acceptance of positive emotions). Positive emotion regulation capacities, however, have yet to be studied specifically as predictors of GAD and PTSD comorbidity.

## 6. The present study

Therefore, the present study examined emotion dysregulation and positive emotion regulation capacities as differentiators of PTSD alone versus PTSD comorbid with GAD. Due to the limited research in this area, hypotheses were based on prior findings of specific deficits in PTSD and in GAD (e.g., Newman et al., 2013) and data related to difficulties with emotional intensity and regulation of emotions (e.g., Mennin et al., 2005) and avoidance behaviors (e.g., Lloyd et al., 2014) faced by those with PTSD and GAD comorbidity. Compared to individuals with PTSD alone, individuals with comorbid PTSD and GAD were hypothesized to demonstrate a) fewer emotion regulation strategies, b) more difficulty with goal-directed behavior, c) lower acceptance of negative emotions, and d) higher awareness of positive emotions.

## 7. Method

### 7.1. Participants

The sample consisted of 292 women recruited to participate in a multi-site randomized controlled trial (RCT) of Skills Training in Inter-personal and Affective Regulation Narrative Therapy (STAIR-NT) vs. treatment as usual (TAU). Participants were recruited from six participating clinical sites and included those referred by clinical providers and those self-referred via advertisements. Women were eligible if they were at least 18 years of age and met DSM-IV diagnostic criteria for PTSD (i.e., a score of > 40 on the Clinician Administered PTSD Scale, CAPS-IV; Blake et al., 1995) related to an interpersonal violence experience about which they had at least one clear trauma memory. They were excluded for: diagnosis of substance dependence or severe substance use disorder; un-medicated psychotic symptoms, mania or bipolar disorder; suicide attempt or self-injurious behavior that required medical attention in the three months prior to enrollment; or current violent romantic relationship. Other comorbidities (e.g., severe depression, moderate substance use, personality disorder, and/or medicated bipolar or psychotic disorders) were not exclusionary. The data for the present study represent a subset of the baseline (pre-intervention) data.

Participants were representative of the public sector settings from which they were recruited. The mean age was 42.57 years (*SD* = 11.92; *range* = 19 – 65). Nearly half (45.7 %) self-identified as White, while 33.0 % identified as African American, 2.7 % as Asian, 1.7 % as Native American, and 16.8 % as another racial group. The majority (80.5 %) identified as non-Hispanic. The majority of the sample (88 %) self-identified as heterosexual/straight, and most were either single (45.2 %) or separated/divorced (34.2 %); 11.6 % were married, 5.1 % living with a significant other, and 2.8 % widowed. The sample was predominantly low-income; nearly half (42.5 %) reported no personal earnings or relying entirely on government assistance. Only 12.6 % of the sample reported earning more than $30,000 per year.

## 8. Measures

Demographic data were collected using a study-created socio-demographics questionnaire. Diagnosis of PTSD, and PTSD symptom severity, were determined using the CAPS-IV (Blake et al., 1995), a well-established, structured clinical interview for the diagnosis of PTSD according to DSM-IV criteria, with excellent inter-rater, test-retest reliability, and convergent and discriminant validity across a range of clinical settings and populations (Weathers et al., 2001). The CAPS assesses diagnostic criteria along with intensity of specific symptoms. The CAPS provides assessors with a total PTSD severity score along with adequate information to make a diagnostic determination for PTSD. GAD diagnosis was established via the Structured Clinical Interview for the DSM-IV (SCID-IV; First et al., 1996), a standard, structured clinical interview. Both CAPS and SCID-IV were conducted by project co-ordinators who were post-baccalaureate and master’s level interviewers who completed training on both structured diagnostic interviews.

Emotion regulation capacity was assessed using the Difficulties in Emotion Regulation Scale (DERS; Gratz & Roemer, 2004), a widely used, 36-item self-report measure of six domains of emotion dysregulation: nonacceptance of emotional responses, difficulty engaging in goal-directed behavior, impulse control difficulties, lack of emotional awareness, limited access to emotion regulation strategies, and lack of emotional clarity. The six subscales have been found to correlate positively with one another, meaning that individuals who struggle in one domain may be more likely to struggle in another. However, factor analysis by the scale’s authors determined the six factors to be distinct constructs, representing unique dimensions of dysregulation. Items are rated on a scale of 1 (*almost never*) to 5 (*almost always*); higher subscale scores indicated more difficulty with emotion regulation in that domain. Good reliability and validity were established in the original sample, and it has been found to perform similarly in men and women and across racial groups (Ritschel et al., 2015). In the current sample, all subscales had acceptable to excellent reliability (Cronbach’s alpha ranged from.78 – .91 across subscales).

Positive emotion regulation was assessed using the Affective Experience Questionnaire, a study-developed measure that was later published as the Hedonic Deficit and Interference Scale (HDIS; Frewen et al., 2012). The HDIS is a 21-item self-report measure that assesses three distinct domains of positive emotion regulation on a scale of 0 (*not at all or never true*) to 10 (*completely true or very frequent*): the extent of positive emotionality (e.g., “Over the past month, would you say that you have experienced feelings of true happiness, cheerfulness, and joy?”), hedonic deficit (e.g., “Would you say that you can’t (you are not able to) experience feelings of true happiness, cheerfulness, and joy, even when you try, and even when good things in your life happen?”), and negative affective interference (e.g., “When positive events happen in your life… do you feel ‘numb’, like you can’t feel emotions and feelings?”) (Frewen et al., 2012). Subscales were scored such that higher scores indicate more capacity for experiencing and regulating positive affect. Frewen et al. (2012) found the subscales to demonstrate excellent reliability (Cronbach’s alphas from.90 to.96), which was the case in the present sample as well (Cronbach’s alphas from.91 to.96 across subscales).

## 9. Procedures

Data for the study came from a multi-site RCT of STAIR-NT (REDACTED). The institutional review board associated with the coordinating study site approved the procedures and informed consent process. Women recruited from each of these sites completed the preintervention assessment interview and were randomly assigned to treatment conditions (i.e., STAIR-NT or TAU); only pre-intervention data were used in the present analyses.

## 10. Data analytic plan

Data from the CAPS and SCID-IV were used to classify participants as members of one of two groups: (1) participants with a diagnosis of PTSD and no GAD comorbidity, and (2) participants with diagnoses of both PTSD and GAD. Using this classification as a categorical grouping variable, multivariate analysis of variance (MANOVA) was used to explore differentiators of diagnostic group. Because inclusion criteria for the larger study required participants to have a CAPS-IV score of 40 or above, no participants had a diagnosis of GAD in the absence of PTSD. Thus, comparisons to a GAD only group were not possible.

Despite unequal sample sizes between the two comparison groups, Levene’s test indicated equality of variance for most emotion dysregulation and positive emotion regulation domains (*p*s ranged from.35 – .96). The exception was for the HDIS subscale of positive emotionality, for which Levene’s test was significant (*F*(1, 269) = 12.45, *p* < .001). Therefore, the Pillai’s Trace statistic was reported for each MANOVA, as it is more robust to violations of the assumption of equal variances. The first MANOVA explored domains of emotion dysregulation (i.e., subscales of the DERS) and a second MANOVA explored domains of positive emotion regulation (i.e., subscales of the HDIS).

## 11. Results

### 11.1. Preliminary analyses

In order to understand relations among study measures, Pearson’s correlations were obtained on total and subscale scores of the DERS and HDIS. A significant, negative correlation was found between DERS and HDIS total scores in the present sample (*r* = −.65, *p* < .001). DERS subscales were significantly and positive correlated with DERS total score, and with one another, to varying degrees; HDIS total and subscales scores were significantly and positively associated with one another; and DERS and HDIS subscale scores were significantly and negatively associated with one another (See Table 1 for full correlation results). Age at the time of baseline assessment was not significantly correlated with either DERS or HDIS total scores (r’s = .001 and.−.06 respectively), nor did the PTSD and PTSD plus GAD groups differ significantly by age (t = −.36, p = .72). Therefore, age was not considered a potential confound and was not accounted for in subsequent analyses.

Of 292 participants, 30 were excluded for missing data on relevant measures. Groups (PTSD only *n* = 182; PTSD & GAD *n* = 80) were compared with respect to PTSD symptom severity (CAPS-IV) and number of additional comorbid diagnoses (SCID-IV). With respect to PTSD symptom severity, *t*-test revealed a significant difference between the two groups (*t_279_* = −5.22, *p* < .001), with those in the comorbidity group reporting somewhat higher PTSD symptoms (*M*_PTSD Alone_ = 13.89, *M*_PTSD_ with _GAD_ = 15.45). With respect to number of additional comorbid diagnoses, *t*-test revealed no difference between the groups (*p* = .53). Mean number of additional diagnoses for both groups was just over three (*M*_PTSD Alone_ = 3.09, *M*_PTSD with GAD_ = 3.27).

## 12. Primary analyses

The first MANOVA comparing participants in each group on domains of emotion dysregulation revealed significant multivariate differences [*F* (6, 255) = 3.49, *p* = .002, Pillai’s trace = .08, *p* = .002]. Specifically, five of the six DERS domains emerged as significant differentiators of the two groups: limited access to emotion regulation strategies [*F*(1, 260) = 18.63, *p <* .001, η_p_^2^ = 0.07], nonacceptance of emotional responses [*F* (1, 260) = 7.09, *p* = .008, η_p_^2^ = 0.03], impulse control difficulties [*F*(1, 260) = 5.57, *p* = .019, η_p_^2^ = 0.03], lack of emotional awareness [*F*(1, 260) = 4.20, *p* = .042, η_p_^2^ = 0.02], and lack of emotional clarity [*F*(1, 260) = 5.88, *p* = .016, ηp^2^ = 0.02]. In all cases, the participants diagnosed with both PTSD and GAD reported higher levels of emotion dysregulation as compared to the participants in the PTSD only group. Difficulty engaging in goal-directed behavior [*F*(1, 260) = 3.61, *p* = .059, η_p_^2^ = 0.01] did not significantly differ between the two groups. See Table 2 for full results, including group means.

The second MANOVA comparing the groups on domains of positive emotion regulation also revealed significant differences [*F*(3, 267) = 8.11, *p* < .001, Pillai’s trace = .08, *p* < .001]. Positive emotionality [*F* (1, 269) = 6.11, *p* = .014, η_p_^2^ = 0.02] was significantly different between groups; participants with a diagnosis of PTSD but no comorbid GAD (*n* = 187) reported more positive emotionality as compared to those with PTSD and GAD (*n* = 84). Negative affective interference [*F*(1, 269) = 21.35, *p* < .001, η_p_^2^ = 0.01] emerged as a differentiator; in this case, those with comorbid PTSD and GAD reported more negative affective interference than the PTSD only group. Hedonic deficit [*F*(1, 269) = 3.18, *p* = .08, η_p_^2^ = 0.07] did not differ significantly between groups. See Table 2.

## 13. Discussion

Though the framework of emotion dysregulation has been helpful for understanding each PTSD and GAD independently, this study represents a first examination of whether domains of emotion dysregulation and/or regulation of positive emotions could improve understanding of PTSD-GAD comorbidity. Five of six emotion dysregulation domains differentiated those with PTSD alone from those with comorbid PTSD and GAD.

## 14. Specific findings

As hypothesized, lower acceptance of emotional responses was found among those with PTSD and GAD as compared to those with PTSD alone. This is consistent with prior findings linking nonacceptance of emotions to GAD diagnosis (Salters-Pedneault et al., 2006), specifically these data indicate that higher nonacceptance of emotions predict GAD comorbidity within other diagnoses such as with social anxiety disorder (Mennin et al., 2009). This finding is important, as it replicates this pattern and highlights this critical regulation domain as an important differentiator of PTSD comorbidity. Contrary to what was predicted, lower awareness of negative emotions and lower emotional clarity also were found among those with a comorbid presentation. However, these findings are in keeping with prior work that found GAD to be a disorder characterized by lower emotional understanding (Mennin et al., 2005; Salters-Pedneault et al., 2006).

Limited access to emotion regulation strategies and difficulty with impulse control were also higher among those with comorbid GAD than those with PTSD alone; this was not anticipated given the well-documented associations between PTSD and both a lack of strategies (Ehring & Quack, 2010; Weiss et al., 2013) and lower impulse control (Tull et al., 2007), thus all participants were expected to be equally deficient in these domains. A lack of emotion regulation strategies has been associated with more severe symptoms across a range of diagnostic categories (e.g., depression, eating disorders, and personality pathology; Sloan et al., 2017), and difficulty with impulse control is linked with PTSD symptom severity specifically (Tull et al., 2007). Therefore, it was important to investigate whether those in the comorbidity group might have reported greater severity of PTSD symptoms and whether this might underlie these findings. This was investigated in the present sample using *t*-tests to compare the two groups (PTSD alone vs. PTSD with GAD) on total PTSD symptom severity assessed via the CAPS (Blake et al., 1995). While *t*-test did reveal a significant difference between the two groups, with those in the comorbidity group reporting somewhat higher PTSD symptoms, the actual difference in group means was relatively small (*M*_d_ = −1.56) and unlikely to be clinically meaningful. In other words, with very similar overall PTSD severity scores, it is less likely that PTSD symptom severity accounts for group differences in lack of regulation strategies and reduced impulse control, or other differentiating domains. Instead, these findings suggest that it is the complexity introduced by an additional diagnosis that is associated with these increased deficits.

One DERS domain did not differ between the two groups: difficulty engaging in goal-directed behavior. It was expected that this deficit would be higher in those with PTSD and GAD comorbid versus PTSD only, particularly given the difficulty in disengaging from worry that can come with GAD symptoms (Mennin et al., 2005; Salters-Pedneault et al., 2006). However, there was a trend toward a difference, which suggests the need for closer attention to the relation between difficulties in goal-directed behavior and diagnostic comorbidity, particularly because all other DERS domains differed between these two groups.

Two domains of positive emotion regulation differentiated PTSD from comorbid PTSD and GAD. Directly counter to expectations, those with PTSD alone reported more positive emotionality than those with PTSD and comorbid GAD. This was surprising, given robust findings that GAD is differentiated from other anxiety disorders, such as social anxiety, by higher awareness of positive emotions (Turk et al., 2005). Similarly, PTSD has been characterized by discomfort with positive emotionality (Frewen et al., 2012) and low acceptance of positive emotions has been associated with PTSD symptom severity (Weiss et al., 2020b). However, a comprehensive review of the literature identifies deficits in positive emotionality as a salient transdiagnostic factor (Carl et al., 2013). While their review does not include studies on PTSD or its comorbidities, it does establish difficulty with positive emotionality as related to a range of mood and anxiety disorders, and thus it may operate similarly with respect to PTSD and comorbid conditions such that more difficulty might relate to diagnostic complexity. Additionally, recent work has found that the link between positive emotionality deficits and PTSD is mediated by higher anxiety sensitivity (Raudales et al., 2021). Thus, the distress associated with the experience of positive emotions that is reported in PTSD might be exacerbated by higher levels of anxiety overall, as might be found in PTSD with comorbid GAD. It is worth noting, however, that this HDIS domain was the sole subscale that violated the assumption of equality of variance for the MANOVA. Therefore, this result may be less robust than the others, and replication will be important for understanding the nature of this relation.

Also inconsistent with predictions was the finding that greater negative affective interference was associated with PTSD and GAD comorbidity. However, as above, this may be explained by the addition of higher anxiety sensitivity that may accompany GAD (Raudales et al., 2021). GAD may both further complicate the experience of positive emotions without distress among those with PTSD and may contribute to the experience of intrusion of negative affect when positive emotions are present. Hedonic deficit did not differentiate the groups in this sample, nor was it anticipated that it would. This domain of regulation differs from the others examined, as this relates more to a lack of, or diminished capacity to experience, positive emotions, rather than distress related to positive affect. In this sample, while the mean hedonic deficit score for the comorbidity group was higher than that of the PTSD only group, variability within each group was high, suggesting that there isn’t a typical degree of hedonic deficit associated with either PTSD or GAD, nor their comorbidity.

Taken together, findings differed from expectations in several ways. Based on prior findings regarding predictors of GAD, of PTSD, and of GAD comorbidity with other anxiety disorders, we expected specific constellations of domains to be more elevated in those with comorbidity, but instead found near universal elevations in difficulties regulating both negative and positive emotions in those with comorbid PTSD and GAD. These results may indicate that, rather than domain specific deficits, it is overall severity of dysregulation that predicts more comorbidity, and perhaps that it is the addition of a general anxiety piece that puts an individual at particular risk for high levels of dysregulation (Raudales et al., 2021).

## 15. Clinical implications

These findings have implications for case conceptualization and treatment planning with trauma-exposed patients who present with comorbid PTSD and GAD. Findings highlight the importance of examining and talking with patients about the function of their GAD symptoms in the context of PTSD symptoms. Patients can be alerted to how worry and other prominent symptoms of GAD may be exacerbated by a hyperfocus on anticipated negative outcomes, and the ways in which it may serve an avoidance function. Specifically, worry may make GAD particularly intractable in patients with trauma histories and PTSD, as these individuals may avoid aversive memories and negative emotions by engaging in present-focused worrying about minor matters (Borkovec et al., 2004). In other words, in accordance with the contrast avoidance model, those with comorbid PTSD and GAD may respond less to interventions due to the tendency to shift focus from the trauma-related material germane to the session to a more general worry in order to reduce overall arousal. In line with this, clinicians could benefit from careful monitoring of people’s capacity for emotion regulation in the context of PTSD, and assess in an ongoing fashion the extent to which heightened dysregulation may be interfering with treatment effectiveness.

## 16. Limitations and future directions

Though it is critical for researchers and clinicians to understand that a PTSD-GAD comorbid presentation is likely to include increased emotion dysregulation across domains, other factors that may be useful in predicting the comorbidity between PTSD and GAD were not accounted for in the present study. For instance, comorbidity of PTSD and GAD has been linked to low perceived social support (Neria et al., 2010) and particular symptom presentations (Kar & Bastia, 2010). Specifically, GAD may be especially associated with PTSD hyperarousal symptoms (Price & van Stole-Cooke, 2015), and thus patients with GAD-PTSD comorbidity may be at risk for chronic autonomic arousal, which can deteriorate physical health and impede normal, adaptive social engagement (Williamson et al., 2015). Anxiety disorders in general are commonly associated with increased activity of the HPA axis and hypercortisolism, whereas PTSD is associated with hypocortisolism, though some specific traumatic experiences like early life adversity may moderate the association between stress and cortisol secretion in adults (see van der Vegt et al., 2010). These factors were not accounted for in the present study. Future studies that examine patterns of cortisol reactivity could better explain when and how emotion dysregulation is heightened for individuals in the comorbid group.

Further, this sample of treatment-seeking patients may evidence different emotion regulation capacities as compared to a non-treatment seeking sample, which may be even more limited in regulatory capacity. The sample was, by design, composed only of women with specific trauma history profiles, and thus generalizability is limited accordingly. This sample did not include men or gender-diverse individuals, whose dysregulation profiles may differ from those evidenced in the present study. There is evidence for gender differences in how dysregulation relates to a wide range of psychopathology (Nolen-Hoeksema, 2012) and to PTSD in particular (Schick et al., 2020). Therefore, the findings of the present study should not be taken to apply to men or gender-diverse individuals. As noted above, replication would also be helpful to verify results regarding positive emotionality in particular, given the potential for these results to be less robust than the others.

Additionally, the absence of a GAD only group precludes the ability to compare the emotion dysregulation profiles of individuals with only GAD vs. those with only PTSD vs. those with the comorbidity. Therefore, while the present study can illuminate what the presence of GAD adds to the PTSD profile, it cannot speculate on what PTSD adds to a GAD-only profile. Future work to examine this additional group would provide a fuller understanding of how these capacities differ across these diagnostic categories and their intersections. Relatedly, though clinicians administering the SCID-IV are instructed not to diagnose GAD if the symptoms exist solely within the context of PTSD (First et al., 1995), PTSD and GAD do share overlapping symptoms (e.g., intrusive worry, irritability, difficulty concentrating), and cases with more symptom overlap may be more likely to receive comorbid diagnoses. The extent to which participants had overlapping symptoms was not accounted for in classifying participants in this study. Future work could examine specific symptom profiles alongside dysregulation domains in order to assess whether controlling for shared symptoms make profiles of dysregulation more of less distinct for those with comorbid conditions.

Despite these limitations, the present study was novel in exploring a common, yet understudied comorbidity in interpersonal violence survivors, using an outpatient clinical sample with significant trauma exposure and symptomatology, and using robust diagnostic measures to determine inclusion into these groups. Exploring specific domains of both emotion dysregulation and positive emotion regulation enhances our understanding of how emotion regulation might be related to the co-occurrence of these two disorders, and these findings can be expanded in future studies that might elucidate further the role of dysregulation in this common comorbidity. In particular, longitudinal studies that systematically examine the temporal relations between the emergence of dysregulation, PTSD symptoms, and GAD symptoms, in relation to trauma exposure, will be critical for determining whether any causal mechanisms may exist. Additionally, whether dysregulation and comorbidity present differently in relation to different forms of trauma exposure could be studied. The present study was limited to women with interpersonal violence exposure, but it could be that exposure to other forms of trauma (e.g., military experience, accidents, natural disasters) prompt differing patterns of dysregulation that relate differently to the PTSD-GAD comorbidity.

Last, future work may use the contrast avoidance model (Borkovec et al., 2004; Newman & Llera, 2011) to better understand the functional role of dysregulation within the PTSD-GAD comorbidity. The pattern that emerged in this study of nonacceptance, nonawareness, and lack of emotional clarity, as characterizing the PTSD-GAD comorbidity may represent strategies to avoid the cognitive and somatic experiences of negative emotions that develop in the aftermath of trauma. Worry, a key feature of GAD, also has been associated with increased hyperarousal and hypervigilance symptoms in PTSD, as it reduces uncertainty about the future (Bardeen et al., 2012). Thus, within this cognitive avoidance framework, GAD in PTSD may reflect the heightened use of this subset of maladaptive regulation strategies to avoid the full impact of trauma-related stimuli. Future work specifically examining the function of dysregulation and worry in individuals with PTSD and comorbid GAD, and how they might interfere with therapeutic interventions for this population, will be critical. Clinical trials would do well to assess symptoms of GAD and domains of emotion regulation as potential mediators and moderators of treatment outcomes and retention. Should they be identified as salient mechanisms in the therapy process, a more systematic incorporation of worry and GAD symptoms into treatment protocols may be warranted

## Figures and Tables

**Table 1:** Descriptive Statistics and Correlations among DERS and HDIS scores.

Scale	*M(SD)*	1.	2.	3.	4.	5.	6.	7.	8.	9.	10.
1. DERS Total	98.26 (23.22)	-	-	-	-	-	-	-	-	-	-
2. HDIS Total	117.68 (40.56)	**−.65**	-	-	-	-	-	-	-	-	-
3. NonAcceptance	16.66 (6.75)	**.75**	**−.41**	-	-	-	-	-	-	-	-
4. Lack Goal Directed Behaviors	16.26 (4.73)	**.69**	**−.32**	**.43**	-	-	-	-	-	-	-
5. Lack Impulse Control	13.66 (5.23)	**.72**	**−.45**	**.37**	**.50**	-	-	-	-	-	-
6. Non Awareness	17.05 (5.13)	**.39**	**−.35**	.12 *	−.06	.04	-	-	-	-	-
7. Limited Strategies	20.95 (7.00)	**.88**	**−.61**	**.64**	**.60**	**.64**	**.18**	-	-	-	-
8. Lack of Clarity	13.39 (4.32)	**.65**	**−.47**	**.30**	**.33**	**.38**	**.45**	**.40**	-	-	-
9. Positive Emotionality	16.57 (11.45)	**−.40**	**.61**	**−.18**	**−.21**	**−.21**	**−.32**	**−.38**	**−.29**	-	-
10. Hedonic Deficit	28.40 (14.16)	**−.40**	**.74**	**−.14**	**−.16**	**−.28**	**−.25**	**−.37**	**−.26**	**.50**	-
11. Negative Affective Interference.	37.36 (26.85)	**.61**	**−.86**	**.46**	**.30**	**.43**	**.27**	**.55**	**.45**	**−.24**	**−.37**

*Note:* Bolded coefficients are significant at *p* < .01;

* *p* < .05

**Table 2:** MANOVA Coefficients and Descriptive Statistics.

F(6, 255) = 3.49, Pillai’s trace = .08, *p* = .002					
Dependent	F	*p*	*η_p_* ^2^	PTSD *M* (SD) N = 182	Comorbid *M* (SD) N = 80
Strategies	18.63	**< .001**	0.067	20.00 (6.73)	23.96 (7.09)
Nonacceptance	7.09	**0.008**	0.027	16.05 (6.77)	18.46 (6.66)
Impulse Control	5.57	**0.019**	0.021	13.21 (5.25)	14.89 (5.36)
Lack of Awareness	4.20	**0.042**	0.016	16.63 (5.16)	18.04 (5.02)
Lack of Clarity	5.88	**0.016**	0.022	13.17 (4.31)	14.56 (4.21)
Goal-Directed Behavior	3.61	0.059	0.014	16.07 (4.69)	17.28 (4.81)
Pos. Emotion Regulation: F(3, 267) = 8.11, Pillai’s trace = .08, *p* < .001					
Dependent	F	*p*	*η_p_* ^2^	PTSD *M* (SD) N = 187	Comorbid *M* (SD) N = 84
Positive Emotionality	6.11	**0.014**	0.022	17.14 (11.85)	13.56 (8.88)
Neg. Affective Interference	21.35	**< .001**	0.012	32.91 (25.82)	48.67 (26.29)
Hedonic Deficit	3.18	0.08	0.074	29.21 (14.33)	25.90 (13.57)

*Note*. Bolded numbers indicate significance below *p* = .05.
